# Time-Dependent Effects of POT1 Knockdown on Proliferation, Tumorigenicity, and HDACi Response of SK-OV3 Ovarian Cancer Cells

**DOI:** 10.1155/2018/7184253

**Published:** 2018-02-06

**Authors:** Hua Zhou, Abdul Mondal, Aleksandra Dakic, Lama Alhawas, Xuefeng Liu, Zhixu He

**Affiliations:** ^1^Department of Obstetrics and Gynecology, Affiliated Hospital of Guizhou Medical University, Guiyang 550004, China; ^2^Department of Pathology, Georgetown University School of Medicine, Washington, DC 20057, USA; ^3^Center for Cell Reprogramming, Georgetown University School of Medicine, Washington, DC 20057, USA; ^4^Center for Tissue Engineering and Stem Cells, Guizhou Medical University, Guiyang 550004, China

## Abstract

The roles of protection of telomeres 1 (POT1) in human ovarian cancer have not been fully elucidated. Here, we investigated the impact of POT1 knockdown (POT1-KD) on in vitro cell proliferation, tumorigenesis, and histone deacetylase inhibitor (HDACi) response in human ovarian cancer-derived SK-OV3 cells. The POT1 gene was knocked down by infection with POT1 lenti-shRNA. POT1, c-Myc, and hTERT mRNA levels and relative telomere length were determined by qRT-PCR; POT1 protein levels were determined by western blot. The relative telomerase activity levels were detected using qTRAP; cell proliferation was assessed using cumulative population doubling (cPD) experiments. Cell tumorigenicity was evaluated by anchorage-independent cell growth assays, and cell response to HDACi was determined by luminescence cell viability assays. Results indicate that lenti-shRNA-mediated POT1-KD significantly reduced POT1 mRNA and protein expression. POT1-KD immediately downregulated c-Myc expression, which led to the inhibition of cell proliferation, tumorigenesis, and HDACi response. However, after brief suppression, c-Myc expression increased in the medium term, which resulted in enhanced cell proliferation, tumorigenesis, and HDACi response in the POT1-KD cells. Furthermore, we discovered that c-Myc regulated cell proliferation and tumorigenesis via hTERT/telomerase/telomere pathway.

## 1. Introduction

Ovarian cancer is the most lethal gynecologic cancer and is the fifth most common cause of malignancy-related death in women [1]. To improve patient outcomes, researchers have focused on elucidating the mechanisms underlying cancer progression and the development of novel cancer therapies. Recently, several reports have shown that depletion of protection of telomeres 1 (POT1) fuels tumorigenesis and leads to cancer development [2, 3]. However, the role of POT1 in the malignant progression of ovarian cancer is unclear.

POT1 is a component of the nucleoprotein complexes that constitute telomeres. Crystal structural analyses have shown that the POT1 protein forms clamps for single-stranded telomeric overhangs and binds these overhangs with exceptionally high sequence specificity [4, 5]. POT1 gene deletions affect telomere structure and function [4]. Previous studies have shown that a reduction in POT1 expression causes cellular senescence and apoptosis [6, 7]. Interestingly, several studies have recently shown that POT1 depletion fuels tumorigenesis and leads to cancer development, increases cancer cell proliferation, and enhances tumorigenicity [2, 3]. However, whether reduced POT1 expression causes cell apoptosis or promotes cell proliferation and exacerbates malignancy in ovarian cancer is unclear.

The proliferation ability and tumorigenicity of tumor cells directly affect tumor progression. c-Myc, which is associated with the malignant progression of cancer [8], binds to the human telomerase reverse transcriptase (hTERT) promoter and positively regulates hTERT to induce telomerase reactivation or to increase telomerase activity [9, 10], each of which leads to telomere lengthening and cell proliferation. In addition, the c-Myc-mediated activation of telomerase triggers chromosome instability (CIN), which results in enhanced tumorigenicity [11, 12]. c-Myc expression is elevated in most human ovarian tumors [13]. However, the way in which c-Myc influences cell proliferation and tumorigenicity in human ovarian cancer POT1-KD cells is unknown.

The treatment of ovarian cancer is difficult for clinicians and researchers who work in the field of oncology. Some targeted therapies are already approved for ovarian cancer among the treatment of primary or recurrent disease, such as antiangiogenic therapy with bevacizumab or PARP inhibitors [14, 15]. Analyses of available data suggest that HDACis exert anticancer effects by specifically targeting transcription factors [16] and promoting deacetylation changes in these nonhistone protein substrates. JNJ-26481585 is a second-generation HDACi, and previous studies have shown that treatment with JNJ-26481585 significantly reduced the growth of rhabdomyosarcoma and lung cancer cells [17, 18]. However, little is known about the effects of JNJ-26481585 on human ovarian cancer cells.

In this study, we aimed to investigate the effects of POT1 gene expression knockdown on in vitro cell proliferation and tumorigenesis in human ovarian cancer SK-OV3 cells and to explore the role of c-Myc in these phenomena. We also investigated whether JNJ-26481585 can effectively treat human ovarian cancer POT1-KD SK-OV3 cells and elucidated the mechanism by which JNJ-26481585 exerts its effects. We hope that this in vitro study can serve as a basis for subsequent in vivo studies and even clinical trials.

## 2. Materials and Methods

### 2.1. Cell Culture and Cell Infection

The SK-OV3 cell line is a hypodiploid human ovary adenocarcinoma cell line and was obtained from the Tissue Culture Shared Resource (TCSR) at the Lombardi Comprehensive Cancer Center (LCCC; Georgetown University, District of Columbia, USA). The cells were cultured in ATCC-formulated McCoy's 5a Modified Medium (Sigma-Aldrich, USA) supplemented with 10% fetal bovine serum (Sigma-Aldrich, USA) and 10 *μ*g/ml gentamicin reagent solution (sc-108071, China). The cells were maintained at 37°C in a humidified incubator with 5% CO_2_ and were passaged at a 1 : 5 ratio when they reached 80–90% confluence. We infected SK-OV3 cells with POT1 lenti-shRNA or nonspecific shRNA (Santa Cruz Biotechnology, USA). Stable clones that expressed the indicated shRNAs were selected with 1.5 *μ*g/ml puromycin dihydrochloride (Gibco, Canada) for 7 days until the control noninfected cells were completely dead. Selection was performed in duplicate.

### 2.2. Cell Proliferation Assay

Cell proliferation was assessed using cPD experiments. Cells were seeded in a 25 cm^2^ flask (Falcon BD, USA) at a density of 50,000–100,000 cells/flask and were cultured in complete growth medium, which was replaced with 5 ml of fresh medium every 2-3 days. When the cells reached 80–100% confluence, the number of cells per flask was determined by a Counter II FL Automated Cell Counter (Thermo Fisher, USA) and the formula *X* = [log⁡⁡10(*N*_H_) − log⁡⁡10(*N*_1_)/log⁡⁡10(2)] [19] was used, where *N*_1_ is the number of live cells in the inoculum (50,000–100,000) and *N*_H_ is the number of cells collected for analysis. To determine the total number of population doublings that the cells had experienced, we measured the population size for each passage and then compared the size of this population to those of the populations for previous passages [20]. To generate growth curves, the total number of days that elapsed between population doublings was plotted against the number of population doublings that occurred.

### 2.3. Quantitative Real-Time PCR (qRT-PCR)

The cells were harvested, and total RNA was extracted using an RNeasy Plus Mini Kit (Qiagen, Germantown, MD, USA) according to the manufacturer's instructions. cDNA was synthesized with 3.0 *μ*g of RNA and a Superscript III kit (Invitrogen, USA) according to the manufacturer's protocol and was stored at −20°C until use. qRT-PCR was performed in a Bio-Rad CFX96 Real-Time System (Bio-Rad, Singapore, USA). All reactions were performed with approximately 50 ng/ml cDNA, which was obtained as described above, and were performed in triplicate. Nuclease-free water was used as a negative control, and *β*-2M was used as an internal control. The following primer sets (Santa Cruz Biotechnology, USA) were used: POT1, 5′-GCTTTGCATCTTTGACGTTTGA-3′ (forward) and 5′-TGTGTGATGTTCAGCCAATGC-3′ (reverse); *β*-2M, 5′-GGACTGGTCTTTCTATCTCTTGT-3′ (forward) and 5′-ACCTCCATGATGCTGCTTAC-3′ (reverse); and c-Myc, 5′-ATGCCCCTCAACGTTAGCTTC-3′ (forward) and 5′-CTGAGACGAGGATGTTTTTGATGAAGG-3′ (reverse). The primers indicated below, which were specific for hTERT mRNA and the TaqMan probe [21], were also used in the qRT-PCR assays (Roche, Indianapolis, IN, USA): forward 5′-TGACACCTCACCTCACCCAC-3′ and reverse 5′-CACTGTCTTCCGCAAGTTCAC-3′ and TaqMan probe 5′-ACCCTGGTCCGAGGTGTCCCTGAG-3′. The relative expression levels of the target genes were estimated using the ΔΔCT method. The results of this experiment were analyzed by the indicated software (Bio-Rad Laboratories, USA).

### 2.4. Western Blot Analysis

The western blot procedure was performed as previously described [22]. GAPDH was used as an internal control. The following antibodies were used (Santa Cruz Biotechnology, USA): anti-POT1 (dilution 1 : 1000), anti-rabbit IgG (dilution 1 : 1000), and anti-GAPDH (dilution 1 : 5000).

### 2.5. Real-Time Quantitative Telomere Repeat Amplification Protocol (qTRAP)

Telomerase activity was analyzed by qTRAP. For this analysis, protein extracts were prepared from the above cells as previously described [23]. SK-OV3 cells infected with the appropriate shRNA were grown in 75 cm^2^ tissue culture flasks (Falcon BD, USA) until they reached 80% confluence, at which time they were lysed. The cell lysates were eventually analyzed via real-time qTRAP, as previously described [23]. The protein concentrations of the cells were determined using the indicated materials (Biotek ELx800, Pierce 660 nm Protein Assay Reagent, Thermo Scientific, USA). A TRAP assay was then performed on 1 *μ*g of protein lysates. For this experiment, the lysates were incubated for 12 h at 33°C in a 40 *μ*l reaction volume containing the following: 1x PCR mix reaction buffer (Invitrogen, USA), 1.5 Mm MgCl_2_, 10 *μ*M dATP, dTTP, dGTP, and dCTP, 0.3 *μ*M telomerase substrate primer (5′-AATCCGTCGAGCAGAGTT-3′, Santa Cruz Biotechnology, USA), and 0.5 *μ*g of T4 gene protein (Amersham Biosciences, USA). Telomerase was inactivated by heating for 10 min at 95°C, and qRT-PCR was performed and the relative number of substrate molecules to which telomeric repeats had been added was calculated. qRT-PCR was performed with SYBR Green (Roche, Indianapolis, IN, USA). Each 25 *μ*l reaction contained 0.3 *μ*M telomerase substrate upstream primer (5′-AATCCGTCGAGCAGAGTT-3′), 0.3 *μ*M telomerase downstream primer (5′-CCCTTACCCTTACCCTTACCCTAA-3′) (the primers were purchased from Santa Cruz Biotechnology, USA), and 1.0 *μ*l of the product from the initial step of the assay. All samples were assayed in triplicate.

### 2.6. Relative Telomere Length and Expression

Genomic DNA was extracted from the cells using a Qiagen DNeasy Blood & Tissue Kit (Qiagen, Germany), and average telomere lengths were determined by a real-time PCR-based telomere assay with modifications [23]. Briefly, the telomere repeat copy number to single gene copy number (*T*/*S*) ratio was determined using a Bio-Rad IQ5 thermocycler with a 96-well platform. Five nanograms of genomic DNA was subjected to PCR reactions with Bio-Rad SYBR Green Super Mixture (Roche, Indianapolis, IN, USA). The following telomere length and HBG1 (a single-copy gene) primers (Santa Cruz Biotechnology, USA) were used for the experiment:

  Tel-1: 5′-CGGTTTGTTTGGGTTTGGGTTTGGGTTTGGGTTTGGGTT-3′
  Tel-2: 5′-GGCTTGCCTTACCCTTACCCTTACCCTTACCCTTACCCT-3′
  HBG1: 5′-TGTGCTGGCCCATCACTTTG-3′
  HBG2: 5′-ACCAGCCACCACTTTCTGATAGG-3′

The reactions comprised the following steps: 1 cycle at 95°C for 5 min, followed by 41 cycles at 95°C for 15 s and 60°C for 45 s. All the samples for both the telomere and the HBG1 reactions were assayed in triplicate, and the *T*/*S* ratio (dCt) for each sample was calculated by normalizing the average HBG1 Ct value to the average telomere Ct value.

### 2.7. Anchorage-Independent Cell Growth Assay

The anchorage-independent growth of cells infected with POT1 lenti-shRNA or empty vector was tested in soft agar, as previously described [24]. A sterile 3% agarose (Sigma, USA) stock solution was prepared in DPBS (Gibco, Canada) and was then diluted by mixing with the complete growth medium appropriate for each cell line. This yielded a 0.6% agarose solution, which was used for the bottom agarose layer, and a 0.3% agarose solution, which was used for the top agarose layer. This experiment was conducted in 12-well tissue culture plates (Falcon BD, USA). Colonies were allowed to form for three weeks, and the media were refreshed once a week during this period. All the experiments were performed in triplicate. The colonies were then stained with 2 ml of 0.1% crystal violet (Sigma-Aldrich, USA) before they were destained by 3 washes in dH_2_O. Images of the colonies were visualized with a digital phase-contrast microscope, and the colonies were counted using an EVOS FL Auto Imaging System and EVOS FL Auto Software (Invitrogen, USA).

### 2.8. Luminescence Cell Viability Assay

**Figure 1:**
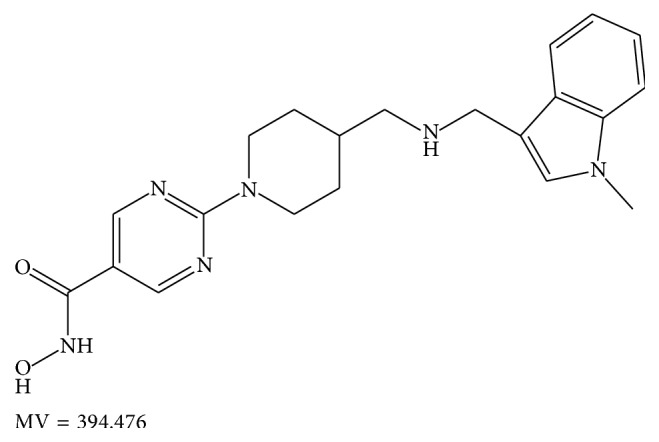
Chemical structure of JNJ-26481585.

SK-OV3 cells were plated in 96-well plates (Falcon BD, USA) at a density of 5000 cells/well and were incubated for 24 h, after which the medium was replaced with a fresh medium containing different concentrations of JNJ-26481585 (concentrations from 0.63 *μ*M to 40 *μ*M were used for this experiment; ApexBio, USA) or the same quantity of dimethylsulfoxide (DMSO, Sigma-Aldrich, USA). The chemical structure of JNJ-26481585 is shown in Figure 1 [18]. The cells were cultured for 48 h before they were subjected to cell viability assays. For this experiment, the plates and their contents were allowed to equilibrate at room temperature for approximately 30 min, after which 50 *μ*l of CellTiter-Glo® Reagent (CellTiter-Glo, USA) was added to each cell culture well. The reagent was also added to control wells containing medium without cells to obtain background luminescence values. The cells were transferred to multiwell plates (Sigma-Aldrich, USA) with opaque walls and then placed on an orbital shaker for 2 min to induce cell lysis. The plates were subsequently incubated for 10 min at room temperature to stabilize the luminescence signals, at which point the luminescence was recorded on a Veritas™ Microplate Luminometer by GloMax® 96 Microplate Luminometer Software (Veritas, USA). Cell viability was determined by calculating the luminescence value ratio (JNJ/DMSO, the viability of JNJ-26481585-treated cells to the viability of DMSO-treated cells).

### 2.9. Statistical Analyses

The results are presented as the mean ± standard deviation, and *P* < 0.05 was considered significant. The levels of significance were defined as follows using two-tailed Student's *t*-tests: ^*∗*^*P* < 0.05, ^*∗∗*^*P* < 0.01, and ^*∗∗∗*^*P* > 0.05 were considered nonsignificant.

## 3. Results

### 3.1. POT1 Knockdown (POT1-KD) Decreased Cell Proliferation and Tumorigenicity in Immediate Effect POT1-KD Cells

**Figure 2:**
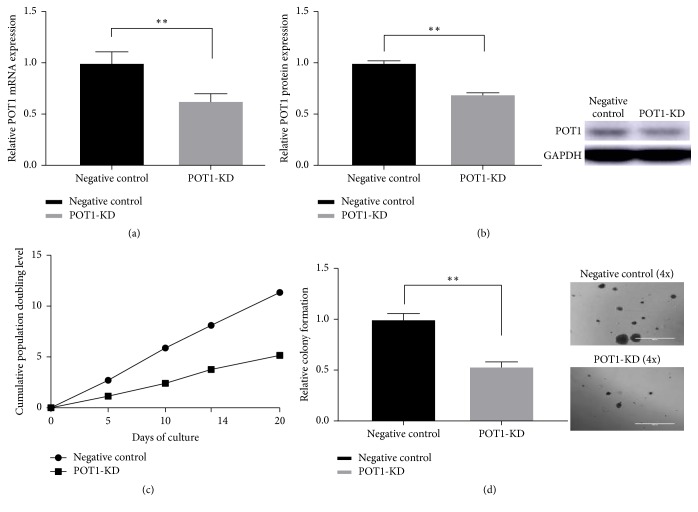
POT1 mRNA expression as determined by qRT-PCR (a) and POT1 protein expression as determined by western blot analysis (b) in shRNA-infected human ovarian cancer SK-OV3 cells. POT1-KD significantly decreased cell proliferation (c) and colony formation (d) in the early POT1-KD SK-OV3 cells. “Negative control” indicates negative control SK-OV3 cells that express a nonspecific shRNA, and “POT1-KD” represents POT1-knockdown SK-OV3 cells that express POT1-specific shRNA. (c) shows the cPDs of the SK-OV3 cells. The cPDs of the negative control cells are depicted with black dots and lines, while the cPDs of the immediate effect POT1-KD cells are depicted with black squares and lines. (d) shows images from the soft agar colony formation assay that used SK-OV3 cells. The dark dots represent the colonies. Images of the negative control cells and the immediate effect POT1-KD cells were captured via digital phase-contrast microscopy at 4x magnification; scale bar: 1000 *μ*m. ^*∗∗*^*P* < 0.01, compared with negative control cells.

Human ovarian cancer SK-OV3 cells were infected with POT1 lenti-shRNA or nonspecific shRNA, the latter of which served as a negative control. We defined the cells that completed the second selection with puromycin as passage 0. To examine the effects of POT1-KD in cells, we determined the POT1 mRNA and protein levels in stably transfected SK-OV3 cells via qRT-PCR and western blot analysis, respectively. The results are expressed as percentages of the controls. POT1 transcript levels in immediate effect POT1-KD cells, which were pre-passage 11 cells and exhibited slower proliferation than that of the negative control cells, were significantly decreased (62.89% versus 100%, ^*∗∗*^*P* < 0.01, compared with negative control cells, Figure 2(a)). The level of POT1 protein in immediate effect POT1-KD cells was also significantly decreased (69.38% versus 100%, ^*∗∗*^*P* < 0.01, compared with negative control cells, Figure 2(b)). The results indicated that we successfully knocked down POT1 gene expression and that POT1-KD SK-OV3 cells stably expressed POT1 shRNA.

To investigate the effect of POT1-KD on cell proliferation, we performed cPD experiments (Figure 2(c)). The data shown represent 5 population doublings. We found that the immediate effect POT1-KD cells proliferated more slowly than the negative control cells. We defined the first population doubling as 0. The cPDs of POT1-KD cells versus negative control cells were 1.15 versus 2.71 in the 2nd cPD, 2.41 versus 5.88 in the 3rd cPD, 3.77 versus 8.11 in the 4th cPD, and 5.15 versus 11.35 in the 5th cPD. In vitro growth curves suggested that the reduction of POT1 expression decreased cell proliferation in immediate effect POT1-KD cells.

The anchorage-independent growth of cancer cells, which is closely correlated with self-renewal and metastasis, reflects the tumorigenicity of these cells [24]. Low-efficiency cell colony formation in soft agar is attributed to mitigated tumorigenicity. To explore the effect of POT1-KD on cell tumorigenicity, we examined the anchorage-independent growth of SK-OV3 cells using a soft agar assay. The cells were plated and cultured in soft agar for 3 weeks. As shown in Figure 2(d), fewer and smaller colonies were formed by immediate effect POT1-KD cells than by negative control cells. The relative colony formation rate of POT1-KD cells was expressed as a percentage of the relative colony formation rate of negative control cells. Compared with the relative colony formation rate in the negative control cells, the relative colony formation rate in immediate effect POT1-KD cells was significantly decreased (53.75% versus 100%, ^*∗∗*^*P* < 0.01). These data indicated that suppressing POT1 expression decreased the tumorigenicity in the immediate effect POT1-KD cells.

### 3.2. POT1 Downregulation Immediately Resulted in a Reduction of SK-OV3 Cell Proliferation and Tumorigenicity via Impairment of c-Myc

**Figure 3:**
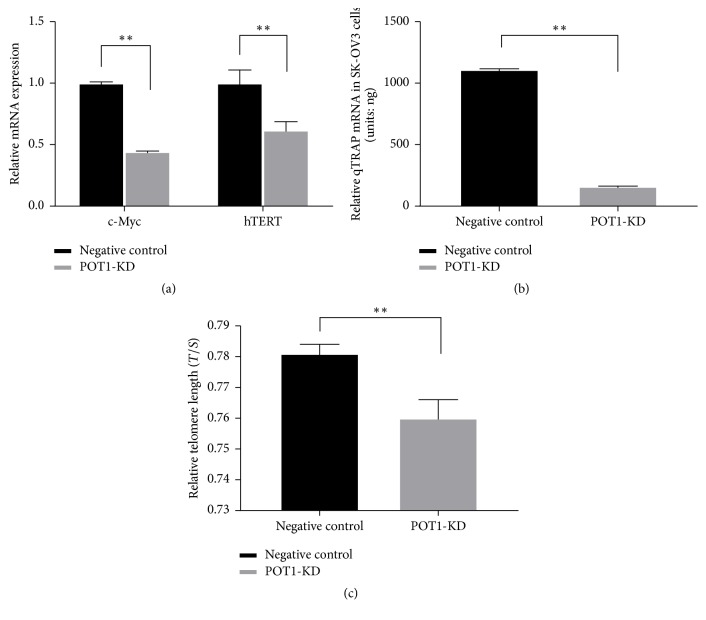
Decreased c-Myc expression resulted in hTERT inhibition and reductions in telomerase activity and telomere length in the immediate effect POT1-KD cells. The mRNA expression levels of c-Myc and hTERT in the immediate effect POT1-KD cells and negative control cells (a); the relative telomerase activity levels in the immediate effect POT1-KD cells and negative control cells (b); and the relative telomere lengths in the immediate effect POT1-KD cells and negative control cells (c). ^*∗∗*^*P* < 0.01, compared with negative control cells.

To elucidate the molecular mechanism that underlies the POT1 downregulation-induced inhibition of cell proliferation and tumorigenicity, we evaluated the transcription levels of c-Myc, which plays a critical role in the proliferation and tumorigenicity of cancer cells [25]. We examined the level of c-Myc mRNA in immediate effect POT1-KD cells via qRT-PCR and expressed the value as a percentage of the negative control. Compared with the mean percentage of c-Myc mRNA expression in the negative control cells, the mean percentage of c-Myc mRNA expression in immediate effect POT1-KD cells was significantly decreased (44.33% versus 100%, ^*∗∗*^*P* < 0.01, Figure 3(a)). These results indicated that POT1-KD decreased c-Myc expression.

hTERT is a downstream gene of c-Myc. The hTERT gene expression levels were determined via qRT-PCR, which showed that hTERT levels were lower in immediate effect POT1-KD cells (61.85% versus 100%, *P* < 0.01) than in the negative control cells (Figure 3(a)). This result indicated that decreases in c-Myc expression resulted in decreases in hTERT mRNA expression in immediate effect POT1-KD cells.

A growing body of evidence indicates that hTERT plays important roles in the promotion of telomerase activity and in telomere maintenance [26, 27]. Relative telomerase activity levels in SK-OV3 cells were detected using the qTRAP assay. The results showed that, compared with the telomerase activity in the negative control cells, the telomerase activity in the immediate effect POT1-KD cells was decreased (160.5 ± 2.1 ng versus 1110 ± 7.1 ng, *P* < 0.01, Figure 3(b)). Telomere length was assessed in SK-OV3 cells using a qRT-PCR-based telomere length assay. The results showed that the telomere length was shorter in the immediate effect POT1-KD cells than that in the negative control cells (0.760 ± 0.006 versus 0.781 ± 0.003, *P* < 0.01, Figure 3(c)).

### 3.3. After Brief Repression, In Vitro Cell Growth Curves Showed That the Proliferation of POT1-KD Cells Increased, and Medium-Term Effect POT1-KD Cells Exhibited More Severe Tumorigenicity Than Their Counterparts

**Figure 4:**
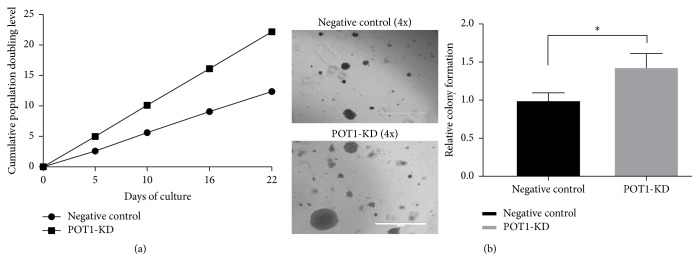
Medium-term effect POT1-KD SK-OV3 cells displayed increased cell proliferation and more severe tumorigenicity than negative control cells. As shown in the cPD experiments, the cPDs of the negative control cells are depicted with black dots and lines, while the cPDs of the medium-term effect POT1-KD cells are depicted with black squares and lines (a). Images of the negative control and the medium-term effect POT1-KD cells were captured via digital phase-contrast microscopy at 4x magnification; scale bar: 1000 *μ*m. The dark dots represent the colonies in soft agar (b). ^*∗*^*P* < 0.05, compared with negative control cells.

cPD experiments were performed again (Figure 4(a)). In these cPD experiments using knockdown and negative control cells, 5 completed population doublings were observed. We defined the first population doubling as 0. The cPDs of POT1-KD cells versus negative control cells were 4.98 versus 2.60 in the 2nd cPD, 10.11 versus 5.62 in the 3rd cPD, 16.12 versus 9.08 in the 4th cPD, and 22.17 versus 12.36 in the 5th cPD. The results of these cPD experiments showed that the POT1-KD cells proliferated more rapidly than negative control cells. Thus, we referred to these POT1-KD cells as “medium-term effect POT1-KD cells.”

Regarding the anchorage-independent growth shown in Figure 3(b), the obtained images showed that medium-term effect POT1-KD cells formed significantly more colonies and larger colonies in soft agar than negative control cells. Compared with the relative colony formation rate in the negative control cells, the relative colony formation rate in the medium-term effect POT1-KD cells was significantly increased (143.52% versus 100%, ^*∗*^*P* < 0.05). These data suggested that, compared with the negative control cells, the medium-term effect POT1-KD cells exhibited enhanced tumorigenicity.

### 3.4. c-Myc Expression Was Significantly Increased, but POT1 Transcription Remained Lower in the Medium-Term Effect POT1-KD Cells Than That in the Negative Control Cells, and Ultimately POT1 Suppression Promoted Cell Proliferation and Tumorigenicity via the Upregulation of c-Myc

**Figure 5:**
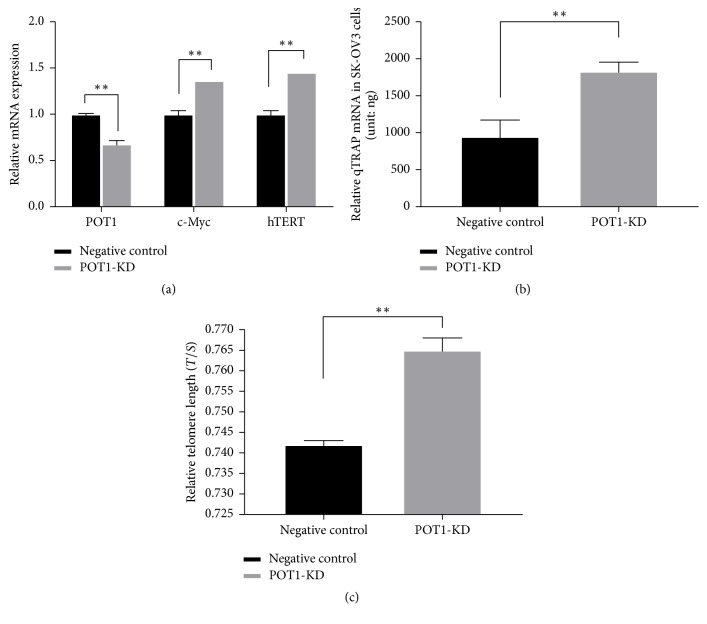
POT1 mRNA expression was reduced, whereas c-Myc mRNA expression was increased in the medium-term effect POT1-KD cells, and as a result of the increase in c-Myc, hTERT mRNA expression was upregulated, telomerase activity was increased, and telomeres were elongated in the medium-term effect POT1-KD cells. The mRNA expression levels of POT1, c-Myc, and hTERT in the medium-term effect POT1-KD cells and negative control cells (a); the relative telomerase activity levels in the medium-term effect POT1-KD cells and negative control cells (b); and the relative telomere lengths in the medium-term effect POT1-KD cells and negative control cells (c). ^*∗∗*^*P* < 0.01, compared with negative control cells.

To assess the efficiency of POT1-KD in the medium-term effect POT1-KD cells, we repeated the qRT-PCR analysis. Compared with the mean percentages of POT1 mRNA expression in the negative control cells, the mean percentages of POT1 mRNA expression in the medium-term effect POT1-KD cells remained significantly decreased (67.78% versus 100%, ^*∗∗*^*P* < 0.01, Figure 5(a)). Conversely, compared with the c-Myc mRNA expression levels in the negative control cells, the c-Myc mRNA expression levels in the medium-term effect POT1-KD cells were significantly increased (136.41% versus 100%, *P* < 0.01, Figure 5(a)).

In conjunction with the increases in c-Myc expression, compared with the hTERT expression in the negative control cells, hTERT expression in the medium-term effect POT1-KD cells was also increased (145.24% versus 100%, *P* < 0.01, Figure 5(a)). This result indicated that POT1 shRNA was still effective in the medium-term effect POT1-KD cells. At the same time, the medium-term effect POT1-KD cells possessed higher telomerase activity, which was nearly 2.0-fold higher (1830 ± 125 ng versus 950 ± 221 ng, *P* < 0.01) than the telomerase activity in negative control cells (Figure 5(b)). Furthermore, compared with the telomere length in the negative control cells, the telomere length in the medium-term effect POT1-KD cells was increased (0.765 ± 0.003 versus 0.742 ± 0.001, *P* < 0.01, Figure 5(c)).

### 3.5. Silencing POT1 Repressed Sensitivity to Anticancer Agent in the Immediate Effect POT1-KD Cells but Ultimately Enhanced the Sensitivity of the Medium-Term Effect POT1-KD Cells to the Antitumor Drug JNJ-26481585, Which Was Likely due to a Different c-Myc Transcriptional Level in the POT1-KD Cells

**Figure 6:**
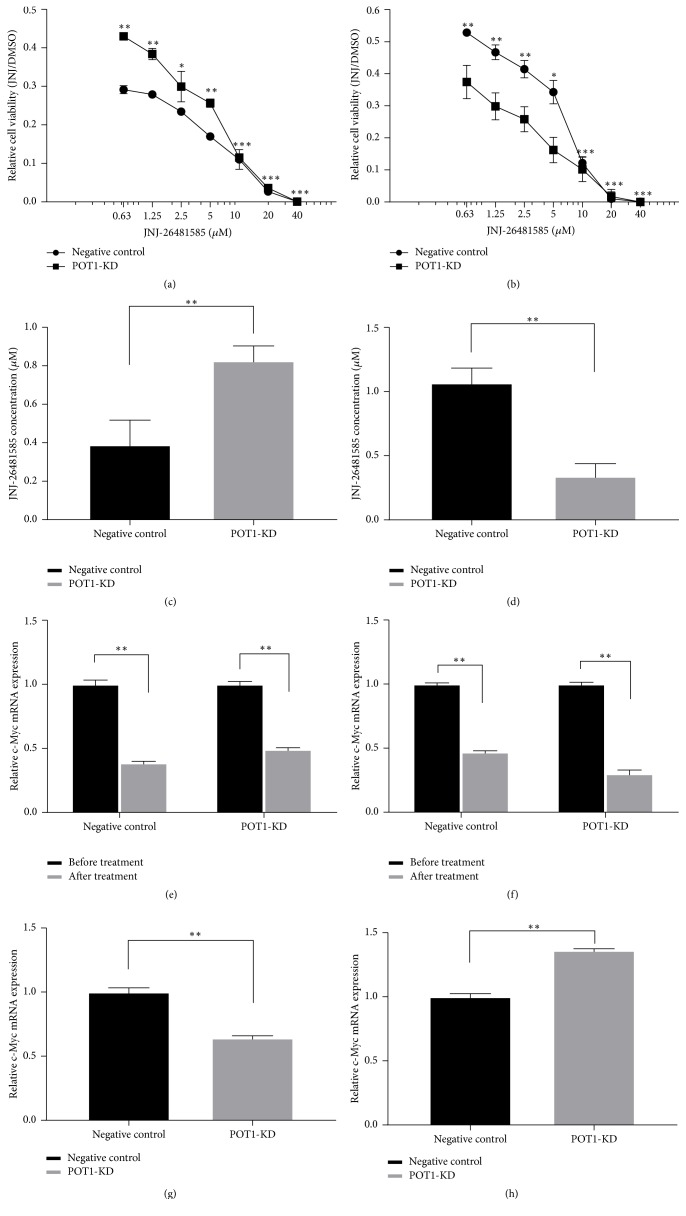
Knockdown of the POT1 gene induced a transient inhibitory effect on the JNJ-26481585 response of SK-OV3 cells, and c-Myc played an important role in the sensitization of SK-OV3 cells to the antitumor agent JNJ-26481585. The relative cell viability of the immediate effect POT1-KD cells and negative control cells (a) and the relative cell viability of the medium-term effect POT1-KD cells and negative control cells (b); the IC50 values of the immediate effect POT1-KD cells and negative control cells (c) and the IC50 values of the medium-term effect POT1-KD cells and negative control cells (d); the c-Myc mRNA expression levels in the immediate effect POT1-KD cells and negative control cells treated with JNJ-26481585 (e); the c-Myc mRNA expression levels in the medium-term effect POT1-KD cells and negative control cells treated with JNJ-26481585 (f); the c-Myc mRNA expression levels in the immediate effect POT1-KD cells and negative control cells before JNJ-26481585 treatment (g); the c-Myc mRNA expression levels in the medium-term effect POT1-KD cells and negative control cells before JNJ-26481585 treatment (h). ^*∗*^*P* < 0.05, ^*∗∗*^*P* < 0.01, and ^*∗∗∗*^*P* > 0.05, compared with negative control cells.

Some reports identified a specific effect of the second-generation histone deacetylase inhibitor JNJ-26481585 to trigger apoptosis in rhabdomyosarcoma and lung cancer cells [17, 18]. In order to address the question of whether JNJ-26481585 can kill SK-OV3 ovarian cancer cells, we tested the effects of JNJ-26481585 alone for the treatment of SK-OV3 ovarian cancer cells. The sensitivity of SK-OV3 cells to JNJ-26481585 was determined by luminescence cell viability assays. SK-OV3 cells were plated in 96-well plates and treated with different concentrations of JNJ-26481585 or the same quantities of DMSO for 48 h. The relative cell viability and the IC50 values for cells served as indices of the sensitivity of POT1-KD cells to JNJ-26481585. Figures 6(a) and 6(b) show that JNJ-26481585 induced SK-OV3 apoptosis in a dose-dependent manner. Cells were treated with JNJ-26481585, the concentration of which varied from 0.63 *μ*M to 5 *μ*M. Compared with the viability of the negative control cells, the viability of the immediate effect POT1-KD cells was significantly enhanced and the viability of the medium-term effect POT1-KD cells was significantly decreased (^*∗∗*^*P* < 0.01 or ^*∗*^*P* < 0.05). However, when cells were treated with JNJ-26481585, the concentration of which varied from 10 *μ*M to 40 *μ*M, there were no statistical differences in the viability of cells between the negative control cells and the POT1-KD cells (^*∗∗∗*^*P* > 0.05). Figures 6(c) and 6(d) show that the IC50 value of the immediate effect POT1-KD cells was increased (0.83 ± 0.08 *μ*M versus 0.39 ± 0.13 *μ*M, ^*∗*^*P* < 0.05) and was approximately 2-fold higher than that in the corresponding negative control cells, while the IC50 value of the medium-term effect POT1-KD cells was decreased (0.34 ± 0.10 *μ*M versus 1.07 ± 0.12 *μ*M, ^*∗*^*P* < 0.05) and was approximately 3-fold lower than that in the corresponding negative control cells. Interestingly, Figures 6(a), 6(b), 6(c), and 6(d) show that the effects of JNJ-26481585-induced cell apoptosis were reduced in the immediate effect POT1-KD cells but that the effects were enhanced in the medium-term effect POT1-KD cells. The data suggested that the immediate effect POT1-KD cells exhibited chemotherapy resistance, while the medium-term effect POT1-KD cells exhibited chemotherapy sensitivity.

To further study the relationship between the cellular response to JNJ-26481585 and the genes involved in intrinsic cell apoptosis, we used qRT-PCR to determine the c-Myc levels, which are expressed as percentages of the corresponding levels in negative control cells. As shown in Figures 6(e) and 6(f), the c-Myc gene expression levels in both groups of negative control cells and in both groups of POT1-KD cells were significantly reduced after JNJ-26481585 treatment compared with before JNJ-26481585 treatment (^*∗∗*^*P* < 0.01). These results suggested that JNJ-26481585 can target the transcription of c-Myc. Before JNJ-26481585 treatment, c-Myc transcription levels in the immediate effect POT1-KD cells were lower (64.18% versus 100%, ^*∗∗*^*P* < 0.01, Figure 6(g)) than those in the corresponding negative control cells. However, compared with the c-Myc gene transcription levels in the corresponding negative control cells, the c-Myc gene transcription levels in the medium-term effect POT1-KD cells were increased (136.22% versus 100%, ^*∗∗*^*P* < 0.01, Figure 6(h)). Taken together, these findings indicated that lower c-Myc expression was closely associated with higher cell viability and resistance to JNJ-26481585 in the immediate effect POT1-KD cells, while higher c-Myc expression was closely associated with lower cell viability and sensitivity to JNJ-26481585 in the medium-term effect POT1-KD cells.

## 4. Discussion

POT1 protein, a single-stranded telomeric DNA-binding protein, plays an important role in telomere protection and in the regulation of telomere length [28]. Previous studies have suggested that the inhibition of POT1 expression is associated with apoptosis or proliferation of tumor cells; however, the role of POT1 in human ovarian cancer remains unclear.

In this study, the data suggested that reduced POT1 expression in the immediate effect POT1-KD cells, which referred to pre-passage 11 cells that exhibited slower growth than that in the control cells, as described previously, caused decreases in cell proliferation and tumorigenicity. These changes were consistent with those noted in a study by Veldman et al. [29]. The POT1 protein can bind to 3′-overhangs in the form of a quadruplex [28, 30], which has been observed in the promoter region of c-Myc [31]. The knockdown of POT1 gene expression changed the spatial conformation of the quadruplex and thus affected the function of the c-Myc promoter, which directly resulted in the downregulation of c-Myc mRNA expression [28, 30, 31]. Subsequently, the transcription of hTERT, a downstream gene of c-Myc [9, 10], was also decreased. As a result, hTERT-dependent telomerase activity was decreased. High telomerase activity triggers CIN and consequently increases tumorigenicity [11, 12]. In this study, impaired c-Myc reduced tumorigenicity via the reduction of telomerase activity in the immediate effect POT1-KD cells. Telomerase maintains telomere length in cancer cells, thereby supporting cell proliferation [32]. Furthermore, reduced telomerase activity causes decreased telomere length, and shortened telomeres contribute to restricted cell proliferation. Thus, the evidence suggested that knockdown of POT1 impaired c-Myc expression, which reduced cell proliferation and tumorigenicity via suppression of the hTERT/telomerase/telomere pathway in the immediate effect POT1-KD cells.

We defined these POT1-KD cells, which proliferated more rapidly than negative control cells, as “medium-term effect POT1-KD cells.” At the same time, as shown in Figure 3, anchorage-independent growth analyses showed that, compared with the negative control cells, the medium-term effect POT1-KD cells exhibited enhanced tumorigenicity. qRT-PCR analysis showed that the expression of POT1 mRNA was significantly reduced in the medium-term effect POT1-KD cells, which indicates that the POT1 shRNA was still effective in these cells and that the increased proliferation and tumorigenicity in the medium-term effect POT1-KD cells were not caused by nonfunctional shRNA-POT1. Contrary to the result of POT1 mRNA expression, c-Myc mRNA transcription levels were significantly increased. Enhanced c-Myc expression in tumor cells occurs via multiple mechanisms, such as gene amplification, chromosomal rearrangement, single-nucleotide mutations, and enhancement of c-Myc protein stability [33–36]. However, the mechanisms that underlie the increase in c-Myc expression noted in this study are unclear and warrant further study. Due to the increased c-Myc transcription, hTERT expression was enhanced, and consequently telomerase activity was promoted and telomeres were extended. In cancer cells, telomere length is determined mainly by telomerase-dependent DNA elongation and erosion, which result from incomplete DNA replication [37]. Thus, when telomerase activity is enhanced in cancer cells, telomere length does not undergo the typical continuous yet slow decrease. Instead, telomere length remains unchanged or increases. In this study, the data indicated that the significantly longer telomeres of the medium-term effect POT1-KD cells compared with those of the negative control cells resulted from increases in telomerase activity. The results of a previous study showed that c-Myc gene transcription was almost undetectable in quiescent cells but was rapidly induced upon mitogenic stimulation [38]; c-Myc then promoted cell proliferation via the hTERT signaling pathway in cancer cells [9, 10]. Our results were similar to those of the indicated studies and suggested that c-Myc upregulation enhanced hTERT pathway-mediated cell proliferation in the medium-term effect POT1-KD cells. High-efficiency cell colony formation in soft agar is attributed to greater tumorigenicity, which is characterized by self-renewal and metastasis [39]. Our data indicated that, in the presence of higher c-Myc expression, hTERT expression was amplified, and telomerase activity was enhanced in the medium-term effect POT1-KD cells. Consequently, the medium-term effect POT1-KD cells produced significantly more colonies and larger colonies in soft agar compared with the negative control cells. These findings suggested that the knockdown of POT1 gene expression ultimately led to increased tumorigenicity via the amplification of c-Myc.

Many studies have indicated that POT1 gene dysfunction in malignant human tumor cells induces senescence and apoptosis [40, 41]. However, recent studies have shown that the repression of POT1 expression can result in tumor progression [2, 3]. In this study, we showed for the first time that reduced POT1 expression in ovarian cancer SK-OV3 cells immediately resulted in temporary inhibition of proliferation and tumorigenicity but ultimately led to enhanced proliferation and tumorigenicity. Furthermore, we found that changes in c-Myc transcription were responsible for these biological phenomena, which occurred via the regulation of the hTERT/telomerase/telomere pathway. Thus, decreased POT1 expression is likely a catastrophic event in ovarian cancer progression because it results in c-Myc dysregulation.

Ovarian cancer is characterized by CIN, a phenomenon that can augment ovarian cancer progression [42]. Our data mentioned above also indicate that POT1-KD enhanced ovarian cancer cell proliferation and oncogenicity through the upregulation of c-Myc. c-Myc is among the most frequently overexpressed genes in human ovarian cancer [43], and it activates the transcription of downstream genes by binding to CACGTG motifs [44]. Therefore, the identification of effective agents that inhibit c-Myc expression is very important to block ovarian cancer progression. However, it is difficult to design inhibitory small molecules that target c-Myc because its protein lacks enzymatic activity and it functions primarily through protein-DNA and protein-protein interactions. Enzymes that catalyze the deacetylation of nucleosomal histones can also modify nonhistone substrates, such as c-Myc, and therefore they are able to regulate c-Myc expression. Several HDACis have already been shown to effectively reduce c-Myc protein levels by directly inducing hyperacetylation and suppressing its transcription [45]. There is growing evidence that JNJ-26481585 alters broad enzyme inhibitory activity in the low nanomolar range and plays a fundamental role in the regulation of c-Myc gene expression governed by epigenetic changes [46]. JNJ-26481585 results in hyperacetylation of c-Myc and subsequent polyubiquitylation and proteasomal degradation of client proteins. This effect is directly related to the proapoptotic activities of the cells [47]. In addition to c-Myc suppression, JNJ-26481585 may have other confounding effects, for example, inducing apoptosis. JNJ-26481585 has been shown to induce morphologic and biochemical changes associated with apoptosis [17, 46, 47]. JNJ-26481585 can elicit diverse biological responses, such as suppression of cell proliferation, induction of cellular differentiation or induction of cellular apoptosis in vitro, inhibition of angiogenesis, and modulation of immune responses in vivo. In therapy studies using a mouse model of B-cell lymphoma, results showed a direct correlation between the induction of tumor cell death by JNJ-26481585 in vitro and therapeutic benefit in vivo [48]. JNJ-26481585, as a novel second-generation HDAC inhibitor, exhibits broad-spectrum antiproliferative activity in solid and hematologic cancer cell lines including ovarian tumor cell lines. This effect was also observed with in vivo ovarian cancer model [49]. In our study, after JNJ-26481585 treatment, c-Myc mRNA expression was significantly downregulated in all treated cells. The data indicated that JNJ-26481585 induced SK-OV3 cell death by targeting c-Myc. Furthermore, in the POT1-KD cells and negative control cells, higher c-Myc expression was always associated with lower cell survival. These findings further confirmed that JNJ-26481585 induced apoptosis in SK-OV3 cells by c-Myc-mediated inhibition. JNJ-26481585 is a promising agent that may be used to arrest ovarian cancer development, and c-Myc is the putative therapeutic target and should be a response predictor for JNJ-26481585 treatment independently of POT1 status. The medium-term effect POT1-KD cells were associated with more severe malignancy, but fortunately amplification of c-Myc enhanced their anticancer activity to JNJ-26481585.

## 5. Conclusions

In summary, our findings indicated that POT1 knockdown resulted in a time-dependent effect on proliferation, tumorigenicity, and HDACi response in SK-OV3 ovarian cancer cells via the regulation of c-Myc. Furthermore, we discovered that c-Myc regulated cell proliferation and tumorigenesis via hTERT/telomerase/telomere pathway.

## Abbreviations

POT1: Protection of telomeres 1
POT1-KD: POT1 knockdown
qRT-PCR: Quantitative real-time PCR
qTRAP: Real-time quantitative telomere repeat amplification protocol
cPD: Cumulative population doubling
hTERT: Human telomerase reverse transcriptase
CIN: Chromosome instability
HDACi: Histone deacetylase inhibitor
DMSO: Dimethylsulfoxide
IC50: Half-maximal inhibitory concentration.
